# High-Intensity Interval Training Is Equivalent to Moderate-Intensity Continuous Training for Short- and Medium-Term Outcomes of Glucose Control, Cardiometabolic Risk, and Microvascular Complication Markers in Men With Type 2 Diabetes

**DOI:** 10.3389/fendo.2018.00475

**Published:** 2018-08-28

**Authors:** Shohn G. Wormgoor, Lance C. Dalleck, Caryn Zinn, Robert Borotkanics, Nigel K. Harris

**Affiliations:** ^1^U-Kinetics Exercise and Wellness Clinic, Faculty of Health and Sciences, School of Applied Sciences and Allied Health, Universal College of Learning, Palmerston North, New Zealand; ^2^Human Potential Centre, Auckland University of Technology, Auckland, New Zealand; ^3^High Altitude Exercise Physiology Program, Western State Colorado University, Gunnison, CO, United States; ^4^Department of Biostatistics and Epidemiology, Faculty of Health and Environmental Sciences, School of Public Health and Psychosocial Studies, Auckland University of Technology, Auckland, New Zealand

**Keywords:** cardiometabolic, microvascular, moderate-intensity continuous training (MICT), high-intensity interval training (HIIT), resistance training, training durability, type 2 diabetes, glucose control

## Abstract

We sought to determine the efficacy of 12 weeks high-intensity interval training (HIIT), compared to moderate-intensity continuous training (MICT) on glucose control, cardiometabolic risk and microvascular complication markers in men living with type 2 diabetes (T2D). Both modalities were combined with resistance training (RT). Additionally, the study aimed to determine the medium-term durability of effects. After a 12-week, thrice weekly, training intervention incorporating either MICT+RT (*n* = 11) or HIIT+RT (*n* = 12), the study concluded with a 6-month follow-up analysis. The middle-aged study participants were obese, had moderate duration T2D and were taking multiple medications including insulin, statins and beta-blockers. Participants, randomized via the method of minimization, performed MICT (progressing to 26-min at 55% maximum estimated workload [eWLmax]) or HIIT (progressing to two variations in which twelve 1-min bouts at 95% eWLmax interspersed with 1-min recovery bouts, alternated with eight 30-s bouts at 120% eWLmax interspersed with 2:15 min recovery bouts) under supervision at an exercise physiology facility. To account for fixed and random effects within the study sample, mixed-effect models were used to determine the significance of change following the intervention and follow-up phases and to evaluate group^*^time interactions. Beyond improvements in aerobic capacity (*P* < 0.001) for both groups, both training modalities elicited similar group^*^time interactions (*P* > 0.05) while experiencing benefits for glycated hemoglobin (HbA1c; *P* = 0.01), subcutaneous adiposity (*P* < 0.001), and heart rate variability (*P* = 0.02) during the 12-week intervention. Adiposity (*P* < 0.001) and aerobic capacity (*P* < 0.001) were significantly maintained in both groups at the 6-month follow-up. In addition, during the intervention, participants in both MICT+RT and HIIT+RT experienced favorable reductions in their medication usage. The study reported the inter-individual variability of change within both groups, the exaggerated acute physiological responses (using exercise termination indicators) that occurred during the interventions as well as the incidence of precautionary respite afforded in such a study sample. To reduce hyperglycaemia, and prevent further deterioration of cardiometabolic risk and microvascular complication markers (in both the short- and medium-term), future strategies that integrate the adoption and maintenance of physical activity as a cornerstone in the treatment of T2M for men should (cognisant of appropriate supervision) include either structured MICT+RT, or HIIT+RT.

Clinical Trials Registration Number: ACTRN12617000582358 http://www.anzctr.org.au/default.aspx

## Introduction

The burden of diabetes is reflected not only in the increasing numbers of people with type 2 diabetes (T2D), but also in the growing number of premature deaths and morbidity due to diabetes (1) with poor glycaemic control being a major source of morbidity and mortality (2, 3). Diabetes alone increases cardiovascular disease, but in the presence of the cardiometabolic risk factors of hypertension, dyslipidaemia, adiposity and systemic inflammation, the risk of cardiovascular events (i.e., stroke, myocardial infarction) is significantly increased (2). In addition, microvascular complications, including cardiac autonomic neuropathy (CAN), diabetic peripheral neuropathy (DPN) and nephropathy, contribute to morbidity, lower-limb amputations and mortality in people with T2D (1, 4, 5). Furthermore, long-term follow-up studies on people living with T2D (6, 7), in which participants received education and lifestyle advice, in conjunction with antihyperglycaemic medication, to either maintain or to intensify glycaemic control reported that long-term intensive glycaemic control did not lead to further long-term benefits with respect to mortality or macrovascular events. Similarly, Wing et al. (8) reporting on the Look AHEAD project, in which participants accumulated ~3 h of moderate-intensity physical activity each week (coupled with a calorie-restricted diet) for a year, concluded that intensive lifestyle interventions focusing on weight loss, counseling and increased physical activity did not reduce the cardiovascular events across all the participating adults with T2D. In the latest position statement of the American Diabetes Association (ADA), the adoption and maintenance of physical activity still remains a vital component in the management of overall health in individuals with T2D (9). Moreover, structured interventions in which participants engage in planned, individualized, and supervised/monitored programmes (10), such as moderate-intensity continuous training (MICT), have been associated with a reduced risk of major microvascular complications and all-cause mortality in people with T2D (11, 12). More recently, the prescription of structured high-intensity interval training (HIIT) has been reported as an alternate therapy in affecting cardiometabolic health (13–15). Likewise, combined training (MICT and resistance training [RT]) has been reported as both comparable to MICT alone (10, 16, 17), and as possessing additional cardiometabolic benefits (9, 18–20). However, only three studies to date have combined resistance exercises with HIIT in people with T2D (21–23); but none of these three studies included a MICT comparative group. Moreover, only one pilot study to date has investigated the effects of HIIT alone on autonomic function (24). In addition, the durability of HIIT-derived benefits is unknown as only one pilot study has reported data beyond the intervention phase (25). Hence, the primary objective of our study was to determine the efficacy of 12-week HIIT combined with RT (HIIT+RT), compared to combined MICT and RT (MICT+RT) on glycated hemoglobin (HbA1c) in men living with T2D. Secondary objectives of the study were to compare the efficacy of the two training modalities on the cardiometabolic risk factors of resting blood pressure, adiposity, high-density lipoprotein cholesterol (HDL), triglycerides (TG) and high sensitivity C-reactive protein (hs-CRP) and microvascular complication markers of heart rate variability HRV distal tactile sensation, postural stability, isokinetic ankle strength and the urine albumin-to-creatinine ratio. The final objective was to determine whether any effects achieved for both training modalities were sustained after a 6-month follow-up phase.

Determining both the short- and medium-term effects on glycaemic control and cardiometabolic risk and microvascular complication markers for two alternate exercise modalities for men with T2D will provide exercise physiologists a better understanding of exercise prescription which will assist with their goal of optimizing patient care. We hypothesized that HIIT+RT would be more effective than MICT+RT in reducing HbA1c during a 12-week supervised exercise intervention in men with T2D.

## Materials and methods

### Study participants

**Figure 1:**
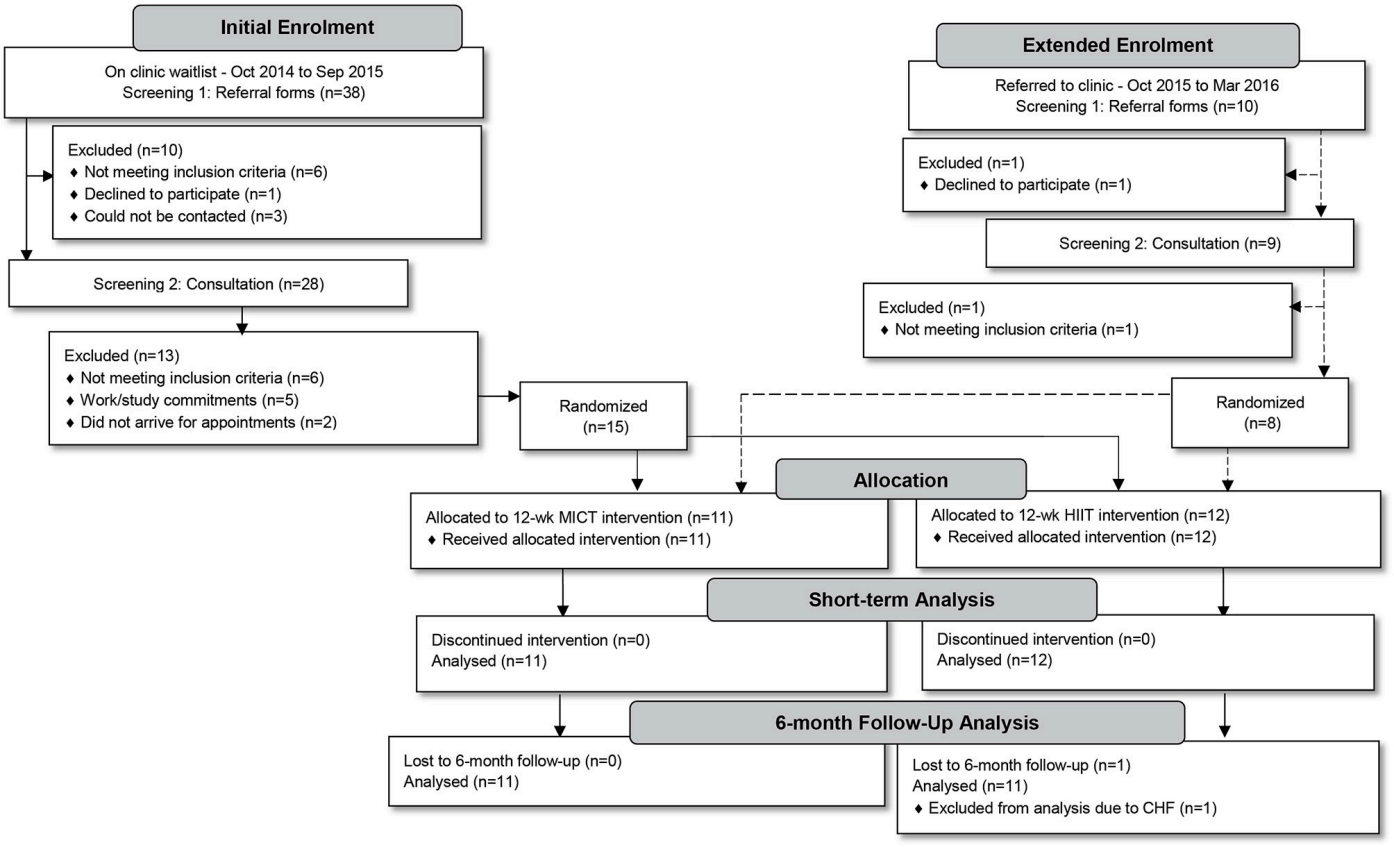
Study participant flow from initial enrolment to medium-term follow-up.

All men living with T2D (aged 35–59 years) referred by their general practitioner to attend a clinical exercise physiology Health and Wellness Clinic (Palmerston North, New Zealand) to participate in a 12-week supervised exercise intervention programme, during the 18 month study-recruitment period, were considered for inclusion (*n* = 48). Potential participants underwent a two-part screening process. Initially, referral forms were screened for the following exclusion criteria: serious recent cardiac, respiratory or musculoskeletal pathologies, neurological disorders (including strokes), unstable proliferative retinopathy, and/or end-stage renal disease. Due to a weight limitation of the cycle ergometers to be used in training, participants exceeding 170 kg were also excluded. Thereafter, potential participants were invited to an individual consultation session in which purposes, risks and benefits of the study protocol were explained. The study protocol was approved by the Auckland University of Technology Ethics Committee (reference no. 14/396), registered as a clinical trial (ACTRN 12617000 582358) and was performed following the tenets of the Declaration of Helsinki. Twenty-three study participants granted their written informed consent and enrolled in the study (Figure 1).

### Study design

Over the 18-month period (using a staggered structure of one or two potential participants per fortnight—as per their referral to the clinic) individual consultations were conducted. Our study was divided into two phases in which all participants were to undergo a 12-week progressive training intervention phase incorporating either MICT+RT or HIIT+RT, after which all participants would complete a 6-month follow-up phase. Sample size calculations were based on a smallest meaningful difference in HbA1c of 3.0 mmol/mol with a standard deviation of effect of 2.5 mmol/mol, α = 0.05, 1–β = 0.80 with the calculation yielding 11–12 participants per group. Recruitment aimed to enroll an additional participant per group to account for a predicted ~10% dropout. However, after an initial 12-month enrolment period only 15 participants had been randomized into the two intervention groups (Figure 1). Similar to prior studies reporting low enrolment rates (26–28), many of our potential participants did not meet study inclusion criteria (*n* = 12). To meet the planned sample size, the enrolment period was extended by 6 months, only enrolling an additional eight participants. The final study sample consisted of 23 participants (MICT+RT, *n* = 11; HIIT+RT, *n* = 12).

### Baseline data

The following data were obtained and used in the intervention phase to randomly allocate participants to groups and were used to guide the initial individualized programme prescription.

#### Referral note details and initial consultation data

During the initial consultation, the medical history and referral note details of age, diabetes duration, ethnicity and current medications were confirmed. All changes to medications during our study phases were recorded. The 2005 adult, long version International Physical Activity Questionnaire (29) was administered to document habitual physical activity along with questionnaires to document physical activity enjoyment [Physical Activity Enjoyment Scale (PACES), (30)]. Although it is conceivable that only those individuals who had an affinity to exercise enrolled in the study, the purpose of the PACES was to monitor the magnitude of change in physical activity enjoyment during the study. Study participants were explained the purpose of PACES and were instructed to complete the form independently and in relation to physical activity in general and not a particular training modality. Additionally, while all participants were instructed not to alter their usual dietary intake, a questionnaire was administered to monitor for any nutritional changes.

#### Nutrition habits

Current “best practice” national nutritional management for diabetes in New Zealand constitutes the Ministry of Health's (MoH) Eating and Activity Guidelines (31); which are to consume a diet moderate-to-high in carbohydrate and low in dietary fat. These guidelines are currently being challenged in the nutrition-related academic and practice arena (32), with the counter, contemporary argument in favor of carbohydrate restriction, with a greater emphasis on dietary fat. This controversy about what constitutes “best practice” in nutrition is an area of debate that needs to be recognized, but is outside the scope of our study. The scores to monitor nutrition changes were interpreted in alignment with the Eating and Activity Guidelines.

Nutrition habits pertaining to the previous 4 weeks were obtained using the 2008/09 New Zealand Adult Nutrition Survey (NZANS) Questionnaire (33). The questionnaire, which establishes the frequency of which various foods and food groups are consumed, was explained to each participant and a blank copy given to participants to complete independently. To objectively record the responses of various nutrition aspects, a novel approach was applied in which the NZANS responses were grouped into five relevant domains (Appendix 1 in Supplementary Material). A novel scoring system was applied as follows: penalty points were allocated to responses that were contrary to the guidelines (with higher penalty points being allocated to responses further away from the guidelines). The general premise of the NZANS is that Check-box 1 represents never (or seldom) with the successive check-boxes increasing in frequency with Check-box 6 representing a frequency of ≥7 times per week (high frequency). Of note, question 24a was added to ascertain the amount of teaspoons of sugar added to hot beverages daily. Participants fully adhering to the guidelines thus received zero penalty points while those not following any guideline within all domains received a maximum of 200 penalty points. The domains of general food quality, fast foods, refined carbohydrates, smoking and alcohol consumption received maximum penalties of 60, 30, 50, 30, and 30, respectively.

#### Blood sampling

After the consultation and 48 h before the baseline physiological assessments, overnight fasted blood samples were collected and analyzed at the local hospital's laboratory. Participants reported to the laboratory at 08:00 h and a blood sample was collected from SP's antecubital vein. HbA1c was measured on a Bio-Rad Hemoglobin Testing System (D-100™, Bio-Rad Laboratories, Inc., Hercules, CA) using high-performance liquid chromatography. HDL and TG were determined using the enzymatic colorimetric method measured on a cobas® 8000 (cobas c702 [AU1], Roche Diagnostics International Ltd, Rotkreuz, Switzerland) chemistry module. The measurement of hs-CRP was performed with a particle enhanced immunoturbidimetric assay measured on a cobas® 8000 (cobas c702 [AU1], Roche Diagnostics International Ltd, Rotkreuz, Switzerland) chemistry module, with hs-CRP estimation by latex turbidimetric method. Urine albumin-to-creatinine ratio (uACR) was measured with a spot urine sample and micro-albuminuria, for men, was defined as 2.5 ≤ uACR <25 mg/mmol (34). The urinary microalbumin was measured on a cobas® 8000 (cobas c502 [AU2], Roche Diagnostics International Ltd, Rotkreuz, Switzerland) chemistry module using an immunoturbidimetric assay.

### Baseline physiological assessments

#### Physiological assessments (session 1)

Subsequent to the blood samples, participants returned to the clinic for the first of two, 60-min assessments (separated by 48 h) where they were instructed to continue taking their medications, to have a light meal based on usual intake, to avoid caffeine 2 h before testing, and to abstain from exercise for the preceding 48 h. The individual assessments (using a staggered structure of one or two participants per fortnight—as per their referral to the clinic) were conducted, over a 15-month period, using the following protocols and in the following sequence.

##### Random blood glucose

A fingertip capillary blood sample was taken on each arrival to the clinic; assessments were postponed if blood glucose concentrations (BG) were <3.5 or >17 mmol/L. Of note, no participant arrived with a low BG for any of the assessments and one participant had a BG of 19.8 mmol/L, but the absence of ketones in his urine was confirmed before subsequent assessments were conducted.

##### Body mass index (BMI)

Body mass was measured using the pre-calibrated HUR- force platform (ALU4, HUR Labs Oy, Tampere, Finland) with participants standing, in short-pants, motionless on the platform. Body mass was recorded to the nearest 10 g. Height was measured using a wall-mounted stadiometer and recorded to the nearest 1.0 mm. BMI was calculated and reported as kg/m^2^.

##### Monofilament test

Participants were assessed for sensation loss, a marker of DPN (35), by their ability to register a sensation of a monofilament when applied, at 10 sites on the soles of both feet, with sufficient pressure to buckle the 10 g filament (36). The sequence and tempo of the measurements were varied to avoid a predictable pattern. Distal tactile sensation was recorded as a score out of 20.

##### Postural stability

The stabiliometry assessment was performed while the participant maintained a quiet barefoot stance on the HUR Labs iBalance^+^ platform (ALU4, HUR Labs Oy, Tampere, Finland). The software received information (sample rate: 100 Hz) about center of pressure motion in the anterior-posterior and medio-lateral directions and the resultant posturogram provided data on trace length (mm). Postural stability was conducted, after calibration checks according to manufacturer's guidelines, for an eyes open (familiarization) and eyes closed (analysis) condition. For each condition participants stood as stable as possible, for 35-s, with only the last 30-s being recorded (37), with their feet in a joined parallel position and arms relaxed at their sides.

##### Resting blood pressure (BP)

The participants lay supine on a plinth for 10-min after which BP was measured at the brachial artery using the auscultatory method at vertical height of the heart. Bicep circumference allowed correct selection of cuff size (large > 34.5 cm > extra-large). The first Korotkoff sound registered the systolic BP (SBP) and the fifth Korotkoff sound was used to register the resting diastolic BP (DBP) (38). The mean of two BP measurements obtained 2 min apart was recorded (39, 40).

##### Deep-breathing heart rate variability (HRV)

The participants were prepared (chest hair shaven and electrode sites cleaned) and connected to a Custo-med (cardio 110, Müller & Sebastiani GmbH, Ottobrunn, Germany) 12-lead electrocardiogram (ECG). HRV using the inspiration-to-expiration ratio of the cardiac rhythm's R-R interval was used as a marker of autonomic function. To complete the testing procedure, participants (still resting in the supine position) were connected to the ECG and coached to perform deep breathing (six breaths per minute) for a full minute. Heart rate (HR) variance was recorded using the longest R-R interval (in milliseconds) during expiration divided by the shortest R-R interval during inspiration. The mean of six of these individual ratios were recorded as the final ratio (41).

##### Graded exercise test (GXT)

The participants performed an incremental cycle ergometer test (Custo-med ergocontrol 3000, Müller & Sebastiani GmbH, Ottobrunn, Germany), monitored with a 12-lead ECG, to ~80% predicted maximum HR reserve (HRR) [i.e., (0.80 × [220-age] – HR_rest_) + HR_rest_] or a rate of perceived exertion (RPE) on Borg's 6–20 scale (42) of ~15 (i.e., “hard”). The cycle test consisted of a 1-min warm-up at 15 Watts (W) followed by three 4-min stages (cycling at 60 revolutions per minute [rpm]). Based on information obtained during the consultation a starting load of 25–50 W was applied and which increased by 20–35 W for each stage. BP, HR, and RPE were recorded for each stage and interpretation of symptom limits were used to optimize safe increments (41). Subsequently, steady state HRs from the final two stages were extrapolated to the predicted maximum HR in order to estimate maximal workload (eWLmax) (43). As the participants were from a clinical population, stage lengths of 4-min were used to help ensure a physiological steady state was met and by using extrapolated values maximal physiological responses are intentionally removed (43). Data obtained from the participant's final two stages of the GXT was used to predict aerobic capacity via maximal oxygen consumption (VO_2_max) (44).

#### Physiological assessments (session 2)

##### Adiposity

Waist circumference was measured according to the International Society for the Advancement of Kinanthropometry (ISAK) guidelines (45) using a non-elastic measuring tape. With the participant standing in the anatomical zero position the waist circumference was measured in a horizontal plane midway between the last rib and the iliac crest. Subcutaneous adiposity was measured indirectly using skinfold thickness, according to ISAK at seven marked skinfold sites (triceps, subscapular, biceps, supraspinale, abdominal, front thigh and medial calf) using a Harpenden skinfold caliper (Baty International, West Sussex, England) and recorded to the nearest 0.1 mm. Subcutaneous adiposity was reported as the sum of the seven measurements.

##### Postural stability

As per the procedures previously described the postural stability tests was repeated in order to enhance reliability. The mean of the trace length measurements (eyes closed on the firm platform) taken during the two assessment sessions was used for analysis.

##### Resting BP

As per the procedure previously described two BP measurements were repeated. The mean of the four recordings taken during the two assessment sessions was used for analysis.

##### Isokinetic ankle strength testing

Before the isokinetic testing to assess distal muscle atrophy (a marker of DPN), participants were instructed to warm-up with a 5-min cycle on an ergometer maintaining 60 rpm at a comfortable resistance. To conclude the warm-up 30-s ankle plantar and dorsi-flexion stretches were performed. Strength assessments were conducted on a Humac NORM (Computer Sports Medicine Inc., Stoughton, MA) isokinetic dynamometer. The dynamometer was pre-calibrated for speed, weight and position following the instructions of the manufacturer. Peak torque recordings, assessing the ankle joint (of the dominant leg) through full plantar-dorsiflexion range, for the concentric action were obtained (38). Absolute isokinetic torque values (gravity-corrected) were recorded as Newton-meters (Nm). The assessment protocol was standardized with respect to patient set-up, familiarization (1 set of 6 progressive repetitions for the 60°/s trials and 1 set of 3 progressive repetitions for the 30°/s trial), rest intervals (30-s after the familiarization trial repetitions and 60-s between the test repetitions) and test repetitions (2 sets of 3 repetitions). The maximum value obtained during the two sets (reciprocal-contractions) at 30°/s was recorded (38). Participants were assessed in the supine position with a chair back angle of 20° and the knee joint fully extended. Thigh straps were used for leg stabilization and participants were instructed to hold the handles while being verbally encouraged to exert maximal effort during the test.

### Grouping

Data from the consultation and baseline physiological assessments were used to assign participants, via the method of minimization (on the basis of HbA1c, age, VO_2_max, BMI, insulin use, and ethnicity), into the two intervention groups (46). Before commencement of the first intervention session, the lead researcher provisionally paired the initial 10 participants as practically as possible (on the basis of HbA1c, age, VO_2_max, BMI, and ethnicity). Subsequently, an exercise physiologist (EP) blinded to the study randomly assigned the five pairs into the MICT and HIIT groups. Thereafter, as consecutive participants joined the study during the next 12 months, they were allocated, by the lead researcher, to the alternate groups via the method of minimization enhancing comparable group characteristics.

### The study phases

#### Intervention phase

As HIIT studies have no standardized training (47) the intervention duration, and frequency and length of the training sessions were in alignment with both the operations of the exercise physiology clinic and the general training variables of the noted studies; specifically three scheduled 1-h sessions per week (Monday, Wednesday, and Friday) for 12 weeks. Each session was a combined cardiovascular (CV) and RT session separated by a BG check. In order to accommodate CV and RT into each session, the protocols of prior studies were emulated (38, 48), whereby shorter variations of both components (in comparison to alone training) were utilized. As per the study design, one group had the CV component of the combined training intervention progress to longer duration MICT training, while the other group progressed to HIIT. Each intervention was designed to ensure equal energy expenditure during the CV component (i.e., isocaloric).

##### Introductory stage

During the 3-week introductory stage the CV component for both groups had 10-min MICT cycling (50% eWLmax, flanked by a 2-min warm-up and 3-min cool-down of 40% eWLmax) with a target RPE of ~13.

##### Intermediate stage

During the 4-week intermediate stage the MICT+RT group was progressed by increasing the training duration to 17:30 min (flanked by a 3-min warm-up and cool-down of 40% eWLmax) and increasing the training intensity to 55% eWLmax; maintaining the target RPE of ~13. During the same intermediate stage the HIIT+RT group progressed to include 3 bouts of 3:30 min at 75% eWLmax, with a target RPE of ~16, interspersed with similar duration recovery bouts at 45% eWLmax (flanked by a 1:30 min warm-up and 2-min cool-down of 30 and 45% eWLmax, respectively) based on prior reported aerobic interval training (AIT) protocols (49, 50).

##### Advanced stage

For the final 5-week advanced stage, the 32-min MICT sessions included training for 26-min at 55% eWLmax with a target peak RPE of ~14 (flanked by a 3-min warm-up and cool-down of 40% eWLmax). The advanced HIIT stage applied one of two variations alternately with each session requiring 28 min to complete. The first variation included twelve 1-min bouts at 95% eWLmax interspersed with 1-min recovery bouts at 40% eWLmax, aligned to the protocols of Little et al. (51) and Gillen et al. (52), with a target peak RPE of ~18 (flanked by a 2:30 min warm-up and cool-down of 60% and 40% eWLmax, respectively). The second variation, a sprint interval training (SIT) session similar to the (53) protocol, included eight 30-s bouts at 120% eWLmax interspersed with 2:15 min recovery bouts at 30% eWLmax (flanked by a 2:30 min warm-up and cool-down of 40% and 25% eWLmax, respectively).

##### Resistance training

Immediately after the CV component a water break and three static stretches (triceps, hamstring, chest) were performed, totalling 5-min, before confirming BG were >3.5 mmol/L in order to commence the RT component. The RT component contained equal exercises and training variables for both groups and maintained a general strength focus with minimal progression or variation during the study intervention. Four compound exercises and an abdominal exercise were completed by the participants using 2 sets of 15 repetitions at 67% of one repetition maximum (1RM) during the introductory stage, 3 sets of 10 repetitions at 75% of 1RM during the intermediate stage and 2 sets of 12 repetitions at 75% of 1RM during the advanced stage. Of note, strength and balance exercises for the ankles were intentionally omitted so as to not confound DPN variables.

TechnoGym cycle ergometers (Bike 700i, TechnoGym: The Wellness Company, Casena, Italy) and four TechnoGym isotonic machines (Selection Line with Isocontrol display, TechnoGym: The Wellness Company, Casena, Italy) were used by the participants during their training sessions. The TechnoGym Wellness System™ (including TechnoGym/Polar T31 HR straps and individualized Wellness System™ Key) were used to control (i.e., time and wattage) and display each prescribed training session and then recorded training data. The training data were downloaded to the Wellness Expert™ via the Wellness System™ Key on conclusion of each session.

To ensure the participants' workload corresponded to the prescribed intensity and reflected any improved aerobic capacity, each participant had the CV component of their training session modified (during the Friday session of Week 4 and Week 8) by simulating the GXT of their assessment (i.e., three 4-min levels with each level increasing by 20–35 W). These data were used to adjust each participant's workload. Similarly, to ensure that the resistance of the isotonic strength exercises were prescribed appropriately according to the strength progressions of each participant, predicted 1RMs (48) were tested and calculated regularly (on the participants' first and last training day and the Monday of Week 5 and 9). On conclusion of this intervention phase (Friday of Week 12) participants repeated the baseline assessments and questionnaires, commencing with blood tests no sooner than the subsequent Monday and no later than the following morning thereafter (Tuesday).

##### Intervention phase supervision

To enhance safety, all participants were screened before the commencement of each training session, via a standardized “pre-session monitoring procedure.” The lead researcher, assisted by three experienced EPs measured participants' physiological parameters of BG, HR, and BP and recorded these values alongside current feelings of well-being and confirmation that medications for the day had been taken as prescribed. The assisting EPs (having relevant post-graduate qualifications), not directly involved in the study design, received pre-intervention training to enhance their ability to capture reliable data pertaining to the requirements of the study. Additionally, the EPs continually monitored each participant's training and recorded peak RPE, BP, and HR on daily training sheets. Midway during the CV component HR and BP were monitored. Moreover, to ensure due care to each participant the talk test was administered during the CV component and, only when deemed appropriate (factoring in HR, BP, verbal and non-verbal responses), precautionary respite was provided by the supervising EP by reducing cycling duration or intensity by 5–10% of cycling time or by 10–20 W, respectively, for MICT. Precautionary respite for HIIT involved reducing the 1-min bout durations by 50–100% for 1–2 of the 12 bouts. Similar to the pre-session monitoring procedure, a post-session monitoring procedure was administered on conclusion of each participant's combined training session.

##### Acute exaggerated responses

To reduce the risk of significant adverse events, indications for terminating exercise testing (54) were used to define exaggerated responses during the sessions (e.g., exercise SBP or DBP approaching 250 or 115 mm Hg, respectively, exercise-induced angina, exercise-induced musculoskeletal strain) along with post-exercise exaggerated responses [e.g., hypoglycaemia (<3.5 mmol/L or feeling symptoms of hypoglycaemia) or post-exercise hypotension (≤90 or ≤60 mm Hg for SBP and DBP, respectively), with feelings of pre-syncope].

#### Follow-up phase

On completion of the 12-week exercise intervention phase all participants were informed that they would be contacted in 6 months' time to confirm their follow-up assessments. In a real-world setting, upon completion of an exercise intervention, people living with T2D are typically advised to continue their training independently. Hence, the purpose of the follow-up phase was to determine the effects of unmonitored, independent training on HbA1c, cardiometabolic risk, and microvascular complication markers. Participants were advised to continue with their training modality only (i.e., MICT group to continue with MICT, and HIIT group to continue with HIIT) and to include RT into their sessions. To this end, study participants were advised to join a local commercial training facility or adopt regular free living cardiovascular activities to replicate their training modality. Additionally, for the RT component, those participants choosing not to join a commercial facility were provided with a home-based RT programme. During this phase participants were independent and could choose to continue their mode of training at an external facility or environment, and received no further support or supervision. At the end of this 6-month phase participants returned to the clinic and completed the baseline assessments and questionnaires. Additional to the study questionnaires, participants were asked to comment on the regularity of maintaining their training during the 6-month independent training phase. A score of 2 was allocated when a month of training was adequately completed (i.e., 2–3 combined sessions per week for 4 weeks), a score of 1 for partial completion and a score of 0 when no training was done that month. Thus, the score range was between 0 (no training done for 6 months) and 12 (6 months of continued training).

### Statistical analyses

The participants' medical history and ethnicity were recorded and descriptive analyses carried out. Normality of data were appraised using Shapiro–Wilk tests. Data are presented as means ± SD where normality of distribution was confirmed. Alternatively, data are presented as median and interquartile range when not normally distributed. To account for fixed and random effects within the clinical study sample, mixed-effect models were used to determine the significance of change (*P* = 0.05) following the intervention and follow-up phases as well as to evaluate the group^*^time interaction effect. Linear models were conducted for normally distributed data. Alternatively, generalized linear mixed models were conducted. Between-group training variables were compared using unpaired, two-tailed student *t*-tests. Data were analyzed using Stata® (Release 14, College Station, TX: StataCorp LLC). The one drop-out during follow-up was left out of the final analysis and not replaced or imputed. Univariate scatterplots (55) have been presented for some data to detail individual nuance.

## Results

Study participants were middle-aged (MICT+RT = 52.5 ± 7.0; HIIT+RT = 52.2 ± 7.1 years; *P* = 0.92) with moderate-duration T2D (MICT = 8.0 ± 6.0; HIIT = 8.5 ± 4.7 years; *P* = 0.83) and presented with poor blood glucose control (MICT = 58.0 [54.5, 68.5]; HIIT = 62.5 [55.5, 66.5 mmol/mol; *P* = 0.57). Participants were using multiple prescribed medications, and were comparable for all measured variables at baseline, including, BMI (class II obesity), and VO_2_max levels (unfit). Referral information, including health history, medication usage and ethnicity, of the male participants are presented in Table 1 with the data for habitual physical activity and nutrition, PACES, and VO_2_max presented in Table 2. Despite the instruction to participants to not alter their habitual physical activity and nutrition, both these showed improvements during the course of the study (Table 2). Though these improvements occurred in both groups to a comparatively similar extent. Of note, both groups begun the study having a positive attitude toward physical activity (PACES) which significantly improved during the intervention and follow-up phases of the study. Both groups experienced similar significant VO_2_max improvements (> 3.9 mL/kg/min) during the intervention which were also significantly sustained at follow-up.

**Table 1:** Health history, baseline medications and ethnicity of the study participants after randomization.

		**MICT+RT (*n* = 11)**	**HIIT+RT (*n* = 12)**	***P*-value**
Health history	Personal history of MI	2 (18.2%)	2 (16.7%)	0.99
	History of stroke	0	0	NC
	History of TIA	1 (9.1%)	0	NC
	History of AO	0	0	NC
	History of PAD	0	0	NC
	Ex-Smokers (>2 years)	5 (45.5%)	5 (41.7%)	0.86
	Smoking (currently)	0	0	NC
	Family history of MI	6 (54.6%)	5 (41.7%)	0.54
	Atrial fibrillation	0	1 (8.3%)	NC
	Neuropathic pain	0	0	NC
	Mild retinopathy	1 (9.1%)	1 (8.3%)	0.99
	OSA requiring CPAP	3 (27.3%)	2 (16.7%)	0.64
	Arthritis	1 (9.1%)	1 (8.3%)	0.99
	Gout	2 (18.2%)	0	NC
	Total hip replacement	1 (9.1%)	0	NC
**MEDICATIONS**				
Antihyperglycaemia	Biguanide (Metformin)	9 (81.8%)	12 (100%)	NC
	Sulfonylureas	5 (45.5%)	3 (25.0%)	0.40
	Exogenous Insulin	4 (36.4%)	5 (41.7%)	0.99
Hypertension	ACE Inhibitors	4 (36.4%)	10 (83.3%)	0.04
	Non ACE Inhibitors	7 (63.6%)	2 (16.7%)	0.04
	Beta Blockers	3 (27.3%)	2 (16.7%)	0.64
	Diuretic	0	2 (16.7%)	NC
Other Cardiac	Statins	5 (45.5%)	9 (75.0%)	0.40
	Anticoagulants	5 (45.5%)	6 (50.0%)	0.83
Respiratory	Bronchodilators	1 (9.1%)	2 (16.7%)	0.99
	Antihistamines	0	2 (16.7%)	NC
	Reflux	1 (9.1%)	0	NC
Other	Anti-depressants	2 (18.2%)	3 (25.0%)	0.99
	Gout	2 (18.2%)	0	NC
	Erectile dysfunction	0	1 (8.3%)	NC
	NSAID	0	1 (8.3%)	NC
	Analgesics	0	1 (8.3%)	NC
Ethnicity	European	9 (81.8%)	9 (75.0%)	0.99
	Māori	1 (9.1%)	2 (16.7%)	0.99
	Asian	1 (9.1%)	1 (8.3%)	0.99

*Data are number of participants (percentage)*.

*ACE, angiotensin converting enzyme; AO, arteriosclerosis obliterans; CPAP, continuous positive airway pressure; HIIT, high-intensity interval training; MI, myocardial infarction; MICT, moderate-intensity continuous training; NC, not calculable; NSAID, non-steroidal anti-inflammatory drug; OSA, obstructive sleep apnoea; PAD, peripheral artery disease; TIA, transient ischaemic attack*.

**Table 2:** Baseline, 12-week intervention and 6-month follow-up comparisons for habitual physical activity (IPAQ), nutrition (NZANS), physical activity enjoyment (PACES), and aerobic capacity (VO_2_max).

	**MICT+RT (*n* = 11)**	**HIIT+RT (*n* = 12)**	**β**	**SE β**	**Z statistic**	***P***	**β Confidence interval**
**IPAQ**							
Moderate METmin/wk	480 (290, 1,435)	690 (380, 1,234)					
12-week intervention	1,185 (320, 2,520)	1,023 (473, 1,474)	435.80	498.44	0.87	0.382	−541.12–1412.72
6-month follow-up	1,650 (960, 3,095)	1,620 (420, 2,050)^a^	1395.48	503.08	2.77	0.006^**^	409.46–2381.50
Group^*^Time interaction			−312.10	665.97	−0.47	0.639	−1617.37–993.18
Total METmin/wk	1,632 ± 1,017	1,808 ± 1,315					
12-week intervention	2,435 ± 1,816	2,371 ± 1,850	802.85	480.32	1.67	0.095	−138.57–1744.26
6-month follow-up	2,951 ± 2,478	2,763 ± 2,591^a^	1399.65	487.19	2.87	0.004^**^	444.76–2354.53
Group^*^Time interaction			−363.46	733.54	−0.50	0.620	−1801.18–1074.25
Sitting hours	76.4 ± 24.1	80.1 ± 21.4					
12-week intervention	75.4 ± 26.4	70.7 ± 24.5	−1.03	4.75	−0.22	0.829	−10.33–8.28
6-month follow-up	72.2 ± 28.8	68.2 ± 23.9 ^a^	−5.14	4.88	−1.05	0.293	−14.7–4.43
Group^*^Time interaction			6.26	12.55	0.50	0.618	−18.34–30.86
**NZANS**							
Refined CHO (/50)	10.0 (4.5, 15.5)	6.0 (1.5, 10.5)					
12-week intervention	8.0 (2.0, 12.0)	6.0 (3.5, 8.0)	−3.55	1.62	−2.19	0.029^*^	−6.72–−0.37
6-month follow-up	10.0 (3.5, 12.0)	6.0 (4.0, 9.0)^a^	−1.07	1.66	−0.65	0.518	−4.32–2.18
Group^*^Time interaction			−5.32	3.25	−1.64	0.102	−11.70–1.05
Total (/200)	52.1 ± 14.6	47.0 ± 16.2					
12-week intervention	44.6 ± 15.4	41.8 ± 14.3	−7.48	2.20	−3.41	0.001^**^	−11.79–−3.18
6-month follow-up	47.4 ± 15.0	42.4 ± 10.9 ^a^	−4.95	2.26	−2.19	0.029^*^	−9.39–−0.51
Group^*^Time interaction			−7.43	7.06	−1.05	0.293	−21.26–6.40
**PACES (/80)**	64.0 (63.5, 72.5)	65.5 (59.0, 77.3)					
12-week intervention	72.0 (69.0, 75.5)	76.5 (73.0, 78.3)	5.27	1.90	2.78	0.006^**^	1.55–9.00
6-month follow-up	71.0 (67.5, 75.5)	(70.0 63.0, 76.5) ^a^	3.96	1.95	2.03	0.042^*^	0.14–7.79
Group^*^Time interaction			−3.11	4.74	−0.66	0.512	−12.41–6.19
**VO**_2_**max**	22.7 ± 5.3	20.4 ± 6.6					
12-week intervention	27.3 ± 5.5	24.3 ± 6.3	4.64	0.74	6.30	<0.001^***^	3.19–6.08
6-month follow-up	25.5 ± 5.1	23.2 ± 6.8 ^a^	3.31	0.76	4.35	<0.001^***^	1.82–4.80
Group^*^Time interaction			−3.85	3.16	−1.22	0.224	−10.05–2.35

*Data are median (interquartile range), means ± standard deviation*.

a n = 11. Statistical significance is reported as follows:

* *P < 0.05*,

** *P < 0.01*,

*** *P < 0.001 for within group analysis*.

*CHO, carbohydrates; HIIT, high-intensity interval training; IPAQ, International Physical Activity Questionnaire; MET, metabolic equivalent; min/wk, minutes per week; MICT, moderate-intensity continuous training; NZANS, New Zealand Adult Nutrition Survey; PACES, Physical Activity Enjoyment Scale; VO_2_max, maximum aerobic capacity (mL/kg/min); (/50), possible 50 maximum; (/80), possible 80 maximum; (/200), possible 200 maximum*.

With regards to adherence, of the possible 36 sessions, the mean number of sessions attended by the MICT+RT and HIIT+RT group were similar (*P* = 0.82) at 32.5 ± 2.5 (90.4 ± 6.8 %) and 32.8 ± 3.6 (91.2 ± 9.9 %), respectively, and the mean energy expenditure per combined (CV and RT) session per participant was similar (*P* = 0.90) with each participant, on average, having expended ~1,040 kcal per week.

**Figure 2:**
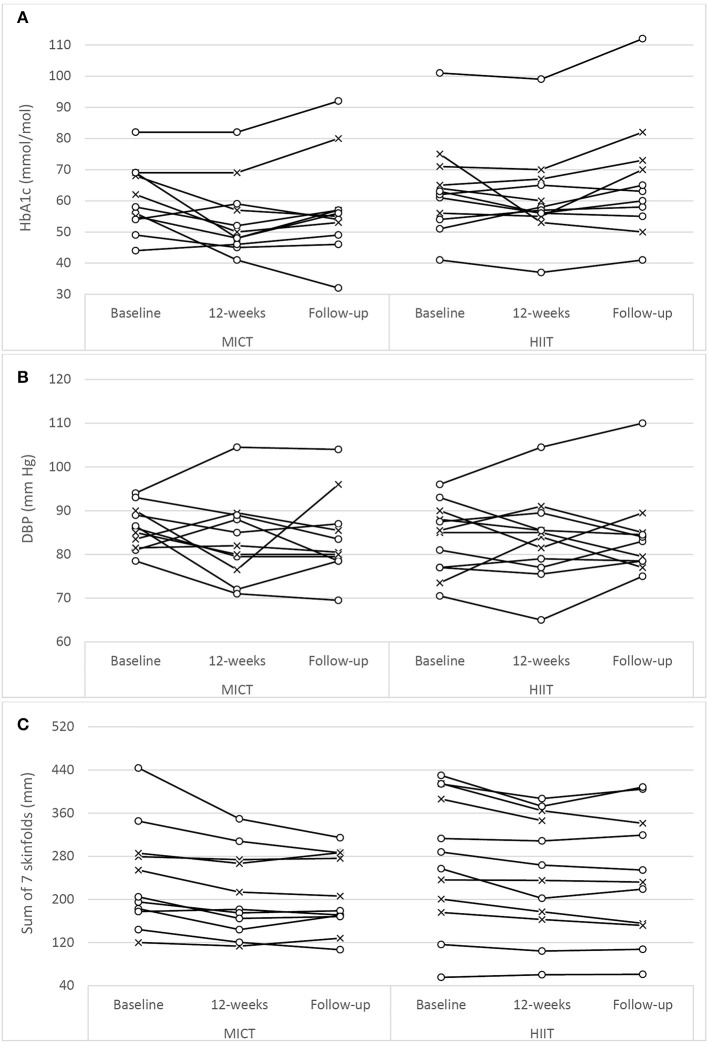
Univariate scatterplots for **(A)** glycated hemoglobin (HbA1c), **(B)** diastolic blood pressure (DBP), and **(C)** subcutaneous adiposity (sum of 7 skinfolds) changes during the study period. MICT, moderate-intensity continuous training; HIIT, high-intensity interval training (o, participants using oral antihyperglycaemic medication; x, participants using exogenous insulin).

**Figure 3:**
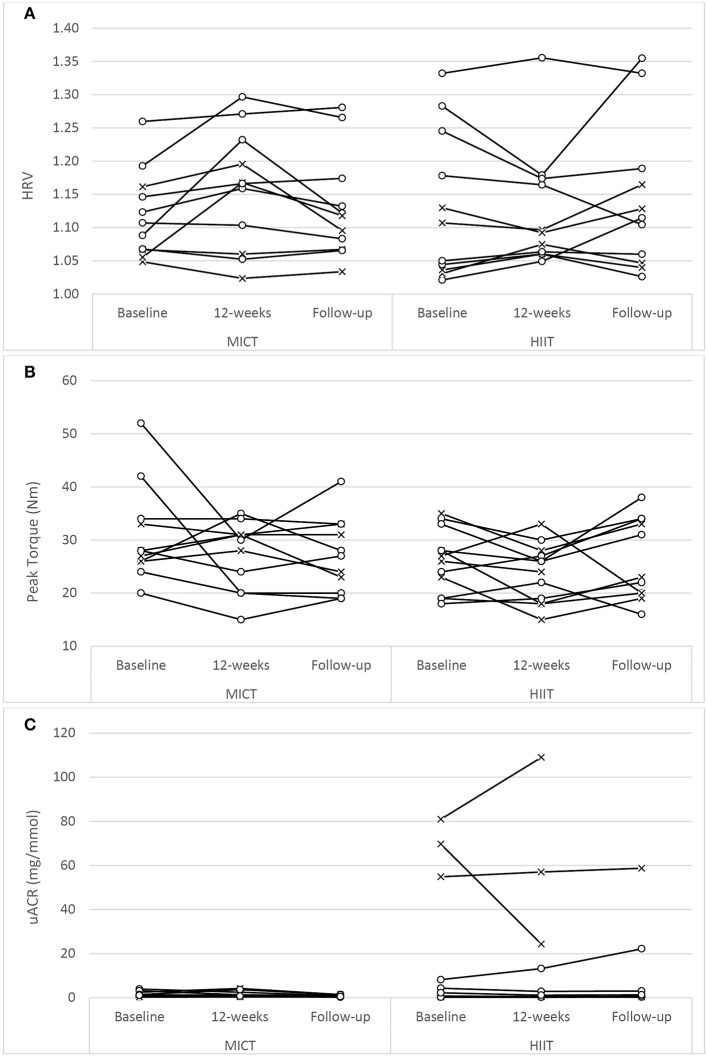
Univariate scatterplots for **(A)** heart rate variability (HRV), **(B)** isokinetic peak torque (dorsiflexion), and **(C)** urine albumin-to-creatinine ratio (uACR) changes during the study period. MICT, moderate-intensity continuous training; HIIT, high-intensity interval training (o, participants using oral antihyperglycaemic medication; x, participants using exogenous insulin).

The assessment results at baseline, on the conclusion of the supervised interventions and the follow-up phase as well as the associated mixed-effects model coefficients, for HbA1c and the cardiometabolic risk markers for BP, adiposity, lipidaemia, and hs-CRP are presented in Table 3. As an example, detailed model coefficients for the mixed-effects model for HbA1c are presented in Table 4. Likewise, the results of the interventions on microvascular complication markers for CAN, DPN, and nephropathy are presented in Table 5 (with the participant with atrial fibrillation being excluded from the analysis regarding HRV)Large inter-individual variations during the study phases were evident with Figures 2, 3 displaying the univariate scatterplots for select cardiometabolic risk (HbA1c, DBP and subcutaneous adiposity) and microvascular complication (HRV, isokinetic dorsiflexion strength and uACR) markers, respectively.

**Table 3:** Baseline, 12-week intervention and 6-month follow-up comparisons for HbA1c and cardiometabolic risk markers.

	**MICT+RT (*n* = 11)**	**HIIT+RT (*n* = 12)**	**β**	**SE β**	**Z statistic**	***P***	**β Confidence interval**
**GLYCAEMIC CONTROL**							
HbA1c	58.0 (54.5, 68.5)	62.5 (55.5, 66.5)					
12-week intervention	50.0 (47.0, 58.0)	57.5 (55.8, 65.5)	−6.27	2.47	−2.54	0.011^*^	−11.11–−1.44
6-month follow-up	55.0 (51.0, 57.0)	63.0 (56.5, 71.5)^a^	−3.60	2.54	−1.42	0.156	−8.59–1.38
Group^*^Time interaction			0.42	7.41	0.06	0.955	−14.11–14.95
**BLOOD PRESSURE**							
SBP	134.8 ± 9.7	133.1 ± 20.9					
12-week intervention	133.2 ± 17.0	133.4 ± 18.1	−1.64	2.92	−0.56	0.576	−7.37–4.10
6-month follow-up	133.4 ± 18.8	131.8 ± 19.6^a^	−2.15	3.00	−0.72	0.474	−8.04–3.74
Group^*^Time interaction			−6.41	7.14	−0.90	0.369	−20.39–7.58
DBP	86.2 ± 5.0	83.7 ± 7.9					
12-week intervention	83.4 ± 9.5	83.6 ± 9.6	−2.82	1.92	−1.47	0.142	−6.58–0.94
6-month follow-up	83.9 ± 9.4	84.1 ± 9.6^a^	−2.56	1.95	−1.31	0.190	−6.39–1.27
Group^*^Time interaction			−5.69	3.32	−1.72	0.086	−12.22–0.81
**ADIPOSITY**							
BMI	35.0 ± 6.1	39.2 ± 9.4					
12-week intervention	34.4 ± 5.5	39.0 ± 9.2	−0.63	0.33	−1.92	0.055	−1.27–0.01
6-month follow-up	34.0 ± 4.6	38.9 ± 9.9^a^	−1.00	0.34	−2.94	0.003^**^	−1.66–−0.33
Group^*^Time interaction			6.23	4.16	1.50	0.134	−1.92–14.38
Waist girth	121.5 ± 15.0	127.4 ± 20.5					
12-week intervention	119.5 ± 14.3	126.9 ± 19.6	−2.02	0.77	−2.63	0.008^**^	−3.52–−0.52
6-month follow-up	119.0 ± 12.6	126.1 ± 21.1^a^	−2.67	0.79	−3.36	0.001^**^	−4.22–−1.11
Group^*^Time interaction			14.79	9.39	1.58	0.115	−3.61–33.18
Skinfolds	239 ± 95	274 ± 123					
12-week intervention	210 ± 79	249 ± 110	−19.31	7.41	−3.96	<0.001^***^	−43.83–−14.79
6-month follow-up	209 ± 71	241 ± 117^a^	−30.98	7.67	−4.04	<0.001^***^	−46.02–−15.94
Group^*^Time interaction			71.48	53.33	1.34	0.180	−33.05–176.00
**BLOOD LIPIDS**							
HDL	1.02 ± 0.26	1.08 ± 0.21					
12-week intervention	1.03 ± 0.25	1.13 ± 0.27	0.01	0.03	0.29	0.768	−0.05–0.07
6-month follow-up	1.01 ± 0.34	1.05 ± 0.22^a^	−0.04	0.03	−1.41	0.158	−0.11–0.02
Group^*^Time interaction			−0.21	0.11	−1.90	0.057	−0.43–0.01
TG	1.7 (1.5, 3.2)	2.2 (1.7, 2.9)					
12-week intervention	1.4 (1.3, 2.0)	1.8 (1.4, 2.4)	−0.79	0.41	−1.92	0.055	−1.61–0.02
6-month follow-up	1.5 (1.2, 3.2)	2.1 (1.4, 2.4)^a^	−0.60	0.41	−1.46	0.144	−1.41–0.21
Group^*^Time interaction			−0.11	2.27	−0.05	0.962	−4.55–4.33
**INFLAMMATION**							
hs-CRP	2.1 (1.6, 5.0)	1.8 (1.3, 3.2)					
12-week intervention	2.6 (1.3, 5.4)	1.8 (1.0, 3.0)	−0.74	0.69	−1.07	0.287	−2.09–0.62
6-month follow-up	3.0 (1.3, 6.6)	2.3 (1.6, 5.5)^a^	−0.60	0.71	−0.84	0.402	−2.00–0.80
Group^*^Time interaction			−3.47	2.91	−1.19	0.233	−9.18–2.24

*Data are means ± standard deviation, median (interquartile range)*.

a n = 11. Statistical significance is reported as follows:

* *P < 0.05*,

** *P < 0.01*,

*** *P < 0.001 for within group analysis*.

*BMI, body mass index (kg/m^2^); DBP, diastolic blood pressure (mm Hg); HbA1c, glycated hemoglobin (mmol/mol); HDL, high-density lipoprotein (mmol/L); HIIT, high-intensity interval training; hs-CRP, high sensitivity C-reactive protein (mg/L); MICT, moderate-intensity continuous training; SBP, systolic blood pressure (mm Hg); TG, triglycerides (mmol/L); Time 1, post 12-week intervention; Time 2, post 6-month follow-up*.

**Table 4:** Effect of training group, time point (TP), age, diabetes duration, ACE inhibitors, use of non-ACE inhibitors, statin use, and those study participants that commenced the study during the extended enrolment period on HbA1c.

**HbA1c**	**β**	**SE β**	**Z statistic**	***P*-value**	**Confidence interval**
Training group	0.42	7.41	0.06	0.955	−14.11–14.95
TP1: Post 12 wk	−6.27	2.47	−2.54	0.011^*^	−11.11–−1.44
TP2: Post 6 mo	−3.60	2.54	−1.42	0.156	−8.59–1.38
Age	−0.42	0.43	−0.98	0.328	−1.26–0.42
T2D duration	0.05	0.54	0.10	0.923	−1.01–1.12
ACE	10.49	6.48	1.62	0.105	−2.21–23.19
Non-ACE	3.42	7.87	0.44	0.663	−12.00–18.84
Statins	0.06	5.75	0.01	0.992	−11.22–11.34
Extended	8.13	5.68	1.43	0.152	−3.01–19.26
Random-effects Parameters	Estimate	SE			Confidence interval
Subject: Identity Var (Cons)	134.68	42.32			72.76–249.33
Var (HbA1c)	33.48	7.12			22.07–50.79

Statistical significance is reported as follows:

* *P < 0.05*.

*ACE, angiotensin converting enzyme inhibitor; Cons, constant; HbA1c, glycated hemoglobin; mo, months; SE, standard error; TP, time point; T2D, type 2 diabetes mellitus; Var, Variable; wk, weeks*.

**Table 5:** Baseline, 12-week intervention and 6-month follow-up comparisons for the microvascular complication markers.

	**MICT (*n* = 11)**	**HIIT (*n* = 12)**	**β**	**SE β**	**Z statistic**	***P***	**β Confidence interval**
**CAN**							
HRV	1.11 (1.07, 1.15)	1.11 (1.04, 1.21)^a^					
12-week intervention	1.17 (1.08, 1.21)	1.09 (1.06, 1.17)^a^	0.05	0.02	2.30	0.021^*^	0.01–0.08
6-month follow-up	1.12 (1.08, 1.15)	1.11 (1.05, 1.18)^a^	0.01	0.02	0.42	0.671	−0.03–0.05
Group^*^Time interaction			−0.03	0.05	−0.61	0.539	−0.13–0.07
**DPN**							
MFT (/20)	18.0 (17.0, 19.5)	18.0 (14.8, 19.3)					
12-week intervention	19.0 (17.0, 19.5)	17.5 (14.5, 20.0)	1.73	0.90	1.93	0.054	−0.03–3.48
6-month follow-up	19.0 (18.0, 20.0)	18.0 (15.0, 20.0) ^a^	2.17	0.91	2.38	0.018^*^	0.38–3.96
Group^*^Time interaction			0.32	1.45	0.22	0.823	−2.51–3.16
Balance (TL)	982 (776, 1,140)	855 (718, 1,021)					
12-week intervention	847 (810, 1,036)	835 (682, 1,041)	−54.34	39.23	−1.39	0.166	−131.23–22.56
6-month follow-up	895 (864, 1,083)	956 (653, 1,157)^a^	−0.70	40.57	−0.02	0.986	−80.21–78.80
Group^*^Time interaction			−127.50	166.04	−0.77	0.443	−452.93–197.93
Dorsi flexion (PT)	30.9 ± 9.1	26.2 ± 5.9					
12-week intervention	27.2 ± 6.5	23.8 ± 5.5	−3.72	1.95	−1.91	0.056	−7.56–0.10
6-month follow-up	27.1 ± 7.0	26.4 ± 7.7 ^a^	−3.94	1.97	−2.00	0.046^*^	−7.80–−0.07
Group^*^Time interaction			1.61	2.69	0.60	0.549	−3.66–6.88
**NEPHROPATHY**							
uACR	1.1 (0.6, 2.0)	1.5 (0.5, 19.9)					
12-week intervention	1.0 (0.7, 1.9)	0.9^*††*^ (0.5, 16.0)	0.06	2.54	0.03	0.980	−4.92–5.05
6-month follow-up	0.9^*††*^ (0.7, 1.3)	0.7 (0.5, 2.6)^b^	−0.49	2.63	0.03	0.851	−5.64–4.65
Group^*^Time interaction			26.28	9.94	2.64	0.008^**^	6.79–45.76

*Data are means ± standard deviation, median (interquartile range)*.

a *n = 11*,

b n = 10. Statistical significance is reported as follows:

* *P < 0.05*,

** *P < 0.01*,

^***^P < 0.001 for within group analysis;

†† P < 0.01, for group^*^time interaction. CAN, cardiac autonomic neuropathy; Dorsi flexion, ankle dorsi-flexion strength; DPN, diabetic peripheral neuropathy; HIIT, high-intensity interval training; HRV, heart rate variability; MFT, 10 g monofilament test; MICT, moderate-intensity continuous training; PT, absolute peak torque (Nm); TL, trace length (mm); (/20), possible 20 maximum; uACR, urine albumin: creatinine ratio (mg/mmol).

### Glycaemic control and cardiometabolic risk markers

Both training progressions produced comparable improvements in HbA1c after the 12-week supervised intervention (*P* < 0.01). However, these improvements were not significantly sustained at the 6-month follow-up (Table 3; Figure 2A). Of note, participant age, T2D duration, hypertension and statin medication usage, or being enrolled during the extended enrolment period did not significantly contribute to the training effect on HbA1c (Table 4). Additionally, the model was repeated to evaluate the effect of background improvements in physical activity and nutrition. Neither the changes to background physical activity (moderate MET min/week) nor nutrition changes (total NZANS penalty score) significantly impacted our findings for HbA1c (*P* = 0.30 and *P* = 0.22, respectively).

Similarly, for waist girth and subcutaneous adiposity there was no difference in the significant reductions experienced by both training modalities during the supervised intervention. Participant age, T2D duration, hypertension and statin medication usage, or being enrolled during the extended enrolment period did not significantly contribute to the training effect on subcutaneous adiposity. Again, changes to background physical activity (moderate MET min/week) and nutrition changes (total NZANS penalty score) did not significantly impact our findings for subcutaneous adiposity (*P* = 0.47 and *P* = 0.67, respectively). Importantly, the reductions to adiposity were the only cardiometabolic risk markers still sustained at the 6-month follow-up (Table 3).

Of note, T2D duration (*P* = 0.001) and the extended enrolment period (*P* ≤ 0.001) did impact the effects of training on DBP. Training modality almost contributed to a significant difference in HDL responses in favor of HIIT+RT (*P* = 0.06) (Table 3) with participant age (*P* = 0.02), T2D duration (*P* ≤ 0.001), non-ACE inhibitors (*P* = 0.02), and statin therapy (*P* = 0.003) also contributing to the effects of training on HDL. Participant age (*P* = 0.02) and statin therapy (*P* < 0.05) were contributing factors to the effects of training on TG and changes to background physical activity and nutrition changes did not significantly impact our findings for TG (*P* = 0.15 and *P* = 0.23, respectively.

### Microvascular complication markers

There was no difference in HRV with respect to MICT+RT or HIIT+RT, with both training modalities producing comparable improvements after the 12-week supervised intervention (*P* = 0.05). However, these improvements were not significantly sustained at the 6-month follow-up (Table 5; Figure 3A). Of note, participant age, T2D duration, hypertension and statin medication usage, or being enrolled during the extended enrolment period did not significantly contribute to the training effect on HRV (Table 5). Similarly, for the 10 g MFT there was no difference in the significant improvements experienced by both training modalities during the supervised intervention. Participant age, hypertension and statin medication usage did not significantly contribute to the training effect on 10 g MFT. T2D duration (*P* = 0.03) and the extended enrolment period (*P* < 0.01) did impact the training effect, and the improvements in this peripheral neuropathy marker were still sustained at the 6-month follow-up (Table 5).

Isokinetic dorsiflexion strength decreased almost significantly (*P* = 0.06), to a comparable extent in both groups, during the supervised intervention. This weakness reached significance (*P* < 0.05) at the follow-up assessment. Of note, participant age (*P* = 0.02), T2D duration (*P* < 0.001), non-ACE inhibitor use (*P* < 0.01), and statin therapy (*P* < 0.001) were contributing factors to the effects of training.

### Acute exaggerated responses

During the completion of the intervention phase there were no major acute adverse events, but a number of acute negative exaggerated responses were recorded in both groups. During the introduction stage (both groups performed low volume MICT prior to that session's RT) precautionary respite had to be administered during 13 of the 184 sessions (7.1%). The cycling intensity had to be reduced once due to elevated exercise BP and five times due to study participants experiencing overexertion (RPE >15). Similarly, the cycling duration had to be reduced twice for overexertion while RT was omitted four times due to low BG readings and once for post-exercise (MICT) hypotension.

During the intermediate stage precautionary respite had to be administered during 14 of the 121 MICT sessions (11.6%) and during 17 of the 132 AIT sessions (12.9%). In the MICT group cycling intensity was reduced seven times for elevated BP, cycling duration and cycling intensity had to be reduced five times and once, respectively, for overexertion (RPE > 15), while RT had to be omitted once due to post-exercise hypotension. In the HIIT group cycling intensity had be reduced thrice for elevated BP, thrice for moderate levels of angina, twice for musculoskeletal discomfort (hamstring strain) and nine times for overexertion (RPE ≥ 17). Additionally, RT had be omitted twice, once for a low BG reading and once for a participant being too “tired” after the cycling.

During the advanced stage of training, precautionary respite had to be administered during 26 of the 165 MICT sessions (15.8%) and during 19 of the 180 HIIT sessions (10.6%). In the MICT group cycling intensity was reduced five times for elevated BP, cycling duration and cycling intensity had to be reduced seven and eight times, respectively, for overexertion, while RT had to be omitted twice for a participant being too tired after the cycling. In the HIIT group cycling intensity had be reduced four times for elevated BP, thrice for moderate levels of angina and 12 times for overexertion (RPE ≥ 19). During this advanced stage no participant experienced a low BG. Of note, the respite was similar for both groups during the intermediate stage (*P* = 0.75) and advanced stage (*P* = 0.15).

## Discussion

The imperative to enhance glycaemic control is compelling as poor control affects the macro and microvascular systems and is a major source of morbidity and mortality in diabetes (2, 3). The present study was designed to determine the glucose control (HbA1c) and markers of cardiometabolic risk and microvascular complication changes induced by a 12-week training programme that progressed either to HIIT+RT or MICT+RT. Moreover, our study included a 6-month follow-up phase to monitor the durability of change.

The improvements in aerobic capacity of our participants are on parity with prior reported improvements in VO_2_max that ranged from 1.9 mL/kg/min (56) to 6.1 mL/kg/min (57) for HIIT. However, unlike our study's MICT+RT improvement of 4.6 mL/kg/min, prior studies have generally reported lower improvements for MICT ranging from 0.2 mL/kg/min (56) to 3.3 mL/kg/min (57).

Whereas three previous studies have, using T2D participants, combined HIIT with RT (21–23), only Praet et al. (23) reported on 1-RM strength and their male-only participants (with clinical signs of neuropathy) experienced significant improvements in upper and lower body strength. Similarly, in our study in which both intervention groups performed the same RT exercises employing the same training variables, significant improvements in strength were achieved in all four isotonic exercises (*P* < 0.01) that were comparable between both groups.

### Intervention phase effects

Our study resulted in a significant reduction in HbA1c that, following the supervised intervention, was similar for both groups (Table 3). This finding is akin to seven of the eight prior HIIT and MICT comparison studies that reported similar improvements in HbA1c for both their intervention groups [(25, 27, 56–60)]. The remaining comparison study Støa et al. (61) reported a greater significant change in HbA1c for HIIT, however, the authors did report that the greater effect may have been due to the significantly higher HbA1c level in their HIIT group (*P* = 0.02) at baseline.

#### Cardiometabolic risk markers

Beyond the positive effects on HbA1c, aerobic capacity, and strength, our study determined the efficacy of MICT+RT and HIIT+RT on the cardiometabolic risk markers of hypertension, adiposity, dyslipidaemia, and inflammation in men with T2D.

In our study there was no interaction effect for either SBP (*P* = 0.37) or DBP (*P* = 0.09) (Table 3), and similar to the large individual variations evident in the univariate scatterplot for DBP (Figure 2B), SBP changes in our study also had disparate changes within the intervention groups. Although prior studies have reported positive results for HIIT's efficacy on BP (22, 24, 26, 28, 56, 57, 62), no study has reported superior benefits over MICT. Interestingly, the only HIIT combined with RT study to report BP change (23) also included only male participants with moderate-duration T2D with similar HbA1c as our participants (but with lower levels of baseline obesity and all using exogenous insulin). As with our study, these authors reported no significant changes to either SBP or DBP. The specific mechanisms responsible for the lack of improvements in BP in our study are not obvious, but possibly the modest weight loss of the obese participants and the impact of their moderate duration of diabetes (Table 5) along with elevated HbA1c could already be contributing to arterial remodeling and stiffening (63). Importantly however, the mean SBP and DBP of both groups did not deteriorate during the 9 month study period.

Although both MICT+RT and HIIT+RT resulted in modest, yet significant, reduction in subcutaneous adiposity the interaction effect in our study was not significant for both waist girth (*P* = 0.11) and sum of seven skinfolds (*P* = 0.18). Similarly, in the 12-week intervention studies that were similar to our study (25, 27, 57, 59, 61), no changes within the groups were reported as being statistically superior. The importance of these initial modest improvement in adiposity following a 12-week intervention must not be overlooked as a *post-hoc* analysis of the Look AHEAD study (64) reported that the overweight and obese T2D participants in their intensive lifestyle intervention group who lost 10% body weight in the first year of the study, had a 20% lower risk of their study's primary outcome (a composite of death from cardiovascular causes, non-fatal acute MI, non-fatal stroke or admission to hospital for angina). Of note, in their *post-hoc* analysis, improvements in fitness, by as much as two metabolic equivalents (7.0 mL/kg/min) in the first year of the study, were not associated with decreases in the risk of the primary outcome. Fat loss addresses the root causes of insulin resistance and is an essential goal for all patients with ectopic lipid deposition, insulin resistance and T2D (65, 66). Recently, in a 4-week study of the effects of MICT and HIIT on non-alcoholic fatty liver disease (in obese adults without T2D) significant reductions in intrahepatic liver content were achieved with only small non-significant changes to body mass (67). As weight loss needs to be significantly higher [closer to 20% of an individual's weight (68)] in people living with T2D wanting to achieve remission, strategies to improve the magnitude of sustained weight loss are needed. Our study demonstrated that fat loss was achieved and maintained for at least 6 months.

In our study both exercise groups, presenting with the characteristic dyslipidaemia of T2D at baseline [consisting of elevated TG [≥1.7 mmol/L] and reduced HDL [≤1.3 mmol/L] concentrations (2, 69)], experienced similar, almost significant improvements in TG (*P* = 0.055) (Table 3). Whereas Alvarez et al. (26) and Mitranun et al. (57) were the only HIIT studies using T2D participants to report significant improvements in HDL, our interaction effect was almost significant (*P* = 0.057) in support of the HIIT group experiencing improved HDL. However, SP age, T2D duration and the use of non-ACE inhibitor and statin medication influenced our finding. Importantly, the lipid profile of both groups were maintained during the study period.

Akin to a recent review on exercise and inflammatory markers in people with T2D (70), the effectiveness of our study's interventions to modify systemic levels of inflammatory markers (e.g., hs-CRP), remains unclear. In parity with our study, the only two studies (23, 25) reporting on hs-CRP changes through HIIT in T2D also used participants with normal levels of hs-CRP (<5 mg/L) at baseline. As inflammatory processes are involved in the pathogenesis of diabetes, and hyperglycaemia itself contributes to the generation of pro-inflammatory factors (70–72), more studies are needed, particularly using participants with elevated hs-CRP, to determine the efficacy of various exercise modalities on systemic inflammation.

#### Microvascular complication markers

Our study was the first to report on the efficacy of MICT+RT and HIIT+RT on microvascular complication markers in men with T2D. Although associated with high morbidity and mortality rates, in its early stages CAN may be completely asymptomatic and detected only by decreased HRV with deep breathing (73). Reduction in HRV is the earliest indicator of CAN (4), with the Sundkvist et al. (74) study being one of the first to report that a reduced variation in the expiration: inspiration ratio of 1.10 (i.e., only a 10% variation) was indicative of autonomic dysfunction. In this early study, people without diabetes (control group), people with diabetes, but without sensory neuropathy, and those with both diabetes and sensory neuropathy had waning ratios of 1.33, 1.27, and 1.16, respectively. In our study both groups significantly improved their HRV during the intervention period (Table 5). The DPN group of the Morrison et al. (75) study in which their participants had similar baseline HRV to our study, and performed MICT (alone) thrice weekly for 12 weeks, reported non-significant improvements in HRV and HbA1c. Parpa et al. (24) did not report on HbA1c, but their participants experienced a significant improvement in HRV following a HIIT pilot study. Restoration of autonomic balance may have clinical importance in preventing adverse cardiovascular events in people with T2D, a concept supported by Vinik et al. (76), who reported that autonomic dysfunction was associated with cardiovascular risk and sudden death and that increased physical activity positively impacts this autonomic imbalance.

DPN is the leading cause of non-traumatic lower-limb amputations (1) and both training groups almost improved significantly (*P* = 0.054) in the 10 g monofilament test, suggesting a possible increase in peripheral sensation. However, concomitantly, both groups experienced an almost significant (*P* = 0.056) counter-intuitive reduction in isokinetic dorsiflexion strength and neither group experienced any change in postural stability (Table 5). T2D duration almost had an influential impact (*P* = 0.051) on the 10 g monofilament findings. Furthermore, SP age, T2D duration and the use of non-ACE inhibitor and statin medication influenced our finding for dorsiflexion strength. Of note, our study intentionally excluded proprioception and lower limb RT so as to limit confounding factors on the DPN findings. Hence, future studies, incorporating such balance and strength exercises are warranted to determine their impact on DPN.

No previous HIIT study has reported on nephropathy measures in participants with T2D. While training modality reportedly contributed to a significant difference in uACR responses (Table 5), it must be noted that the HIIT group was the only group that contained participants with elevated readings at baseline (Figure 3C) and, as such, this finding must be interpreted with caution.

Although the scope of microvascular complications seems vast, not all cell populations are prone to complications with those affected being limited to vascular endothelia, renal mesangial and proximal tubular cells, glomerular epithelial cells, neurons, and glial cells. In these tissues, facilitated diffusion of glucose occurs in an insulin-independent manner via the glucose transporter 1 (GLUT1) with the resultant intracellular hyperglycaemia possibly a key initiating factor in the development of diabetic complications (77). Even though our study did not directly measure these complex mechanisms, the enhanced reduction in overall systemic hyperglycaemia, in which the frequent metabolic demands within the skeletal muscles possibly enhanced glucose transporter 4 (GLUT4) expression, potentially deceased the diffusion of glucose via GLUT1 into the microvascular complication prone cells, thereby resulting in the favorable effects in HRV and the 10 g monofilament test. Further research is however warranted to elucidate the precise mechanism affected by various modes of exercise.

#### Medication changes

As medication, along with habitual physical activity and nutrition changes, confound exercise's efficacy on HbA1c, it is worthwhile to highlight the medication changes that occurred during the study. During the 12-week intervention, both groups had changes to medication usage with the majority being antihyperglycaemic decreases. MICT+RT had one participant no longer on any antihyperglycaemic medication (i.e., partial remission), two participants made minor reductions (~2.0 units) to insulin, one participant took 50% less metformin and sulfonylurea and one participant reduced his BP medication. Similarly, with HIIT+RT all five participants on insulin made reductions (~4.2 units), four participants each took one less metformin with a meal, one participant stopped taking sulfonylureas while one participant increased his sulfonylurea dose. These reductions in medication usage (including insulin) experienced in both training groups are an important finding of our study and, although these reductions confound the comparison of MICT+RT with HIIT+RT, they demonstrate the benefit of regular structured exercise.

#### Training variables

Although our study was the first RCT to determine the efficacy of modalities that combined RT with either HIIT or MICT, in order to minimize confounding factors between the HIIT and MICT group, the RT variables in both groups were equivalent. Notwithstanding the RT component, our study was similar to prior studies comparing HIIT and MICT in people with T2D with regards to the 12-week intervention duration (25, 27, 57, 61) and the frequency of three HIIT sessions per week (25, 27, 56, 57, 61). Although the diverse application of the HIIT variables (i.e., intensity, duration and number of bouts) across all studies limit direct comparisons, our study's HIIT variables were comparable. However, apart from the 10 min SIT session of Ruffino et al. (56) incorporating two 20-s sprints, and the 20 min SIT session of Maillard et al. (60) incorporating sixty 8-s sprints, our study's progressed 28 min sessions were shorter than the progressed AIT sessions that were either 40 min (25, 57), 50 min (61), or 60 min (27, 58, 59) in duration. The durations of the AIT sessions of prior studies are substantially longer than our study and may contribute to the findings reported in these HIIT studies. However, with regards to MICT, only (60) reported that MICT was conducted exclusively on a cycle-ergometer and while (59) reported alternating between cycle-ergometry and treadmill walking, the remaining studies either did not report a specific MICT mode (27, 25) or reported MICT being conducted via a walking mode (56–58, 61). Notably in our study, participants reported a mean RPE during the progressed MICT of 13.6 ± 0.9, whereas in the walking MICT group of Ruffino et al. (56), the only comparative study to report RPE, their participants reported their RPE as 12 ± 1. Of note, as per our study's design, the mean RPE of the HIIT group during the progressed stage (15.2 ± 1.2) was significantly higher than the MICT group (*P* = 0.003). Interestingly, our participants in the HIIT group reported a higher RPE (*P* = 0.009) following the twelve 1-min bouts (16.0 ± 1.5) compared to the eight 30-s higher intensity bouts (14.4 ± 1.2).

Physiologically, during exercise, the coordinated increases in skeletal muscle blood flow, capillary recruitment and GLUT4 translocation (from their intracellular sites to the sarcolemma and T-tubules) and regulation of AMP-activated protein kinase and liver kinase B1 (78), enhance glucose uptake and oxidation (79). Moreover, the latest position statement by the ADA (9) reports that the increases in insulin sensitivity and improved glycaemic control, for both MICT and HIIT, are related to the increased muscle capillary density and increased skeletal muscle oxidative function achieved through the improvements in aerobic capacity. Additionally, regular exercise training improves muscle capillary density, lipid metabolism (9), mitochondrial function, increases mitochondrial biogenesis and increases the expression of GLUT4 (78). However, total training volume *per se*, contributes to the training-induced glycaemic benefits in participants with T2D (80, 81).

Skeletal muscle is a highly dynamic tissue which is composed of individual muscle fibers with a dynamic range of chemical, biomechanical, and physiological properties. The presence of diverse fiber types with distinct ranges of adaptability reflects muscle plasticity to various metabolic and functional demands (82, 83). In people with T2D there is an increased proportion of fast glycolytic fibers (type IIb) and a reduced proportion of slow oxidative fibers (type I) and is the reason people with T2D present with a diminished oxidative capacity (84). Whereas the amount of respite administered for overexertion in all study phases of our study may be due to the indirect estimation of eWLmax, exercise intolerance of this study sample may also have been a factor. Typically, in healthy individuals during MICT, type I fibers are predominantly recruited and during more intense training (i.e., RT and HIIT) the recruitment is predominantly type II fibers. Recent studies have examined the alterations in muscle fiber proportions following medium-term training using MICT, HIIT and/or RT, but such interventions have been conducted in non-diabetic populations (85–87). Although (88) reported that exercise-stimulated skeletal muscle fiber-type alters from type IIb to type I and type IIa (intermediate fibers), without further study the precise effect of MICT, HIIT and/or RT on muscle fiber recruitment, and subsequent adaptation, in people living with T2D will remain speculative.

#### Inter-individual variability

For each dependent variable there were unique participant responses within each group (Figures 2, 3). Although the underlying molecular pathomechanics for sub-optimal responsiveness following various interventions are not yet known, a possible explanation for such individuality in responsiveness could, in part, be genetic susceptibility (89, 90), the complex pathology of the disease itself (77), pre-training muscle fiber characteristics (87) and/or the side effects of the multiple medications, including insulin, statins, antihypertensive and antidepressant agents (91). However, of note, after the preparatory phases of the study design, the progressed sessions were only administered for 5 weeks and, as such, the responsiveness within participants may have been limited and future studies could consider longer intervention durations (e.g., 18 weeks). In addition, limiting the combined sessions to three 1-h sessions per week (as common in many commercial EP settings) may have impacted participant responsiveness as both the CV and RT component were limited to ~30- and ~20-min, respectively. Increasing training frequency and/or training time for either, or both, training components may result in enhanced responsiveness. Anecdotally, and encouragingly, there seemed to be no visual differences in overall changes for the participants who were exogenous insulin users compared to those only on oral antihyperglycaemic medications (Figures 2, 3). Additionally, inspection of individual participant data indicated that unresponsiveness was not consistent across variables. For example, the four participants in the MICT+RT group (and four participants in HIIT+RT) that either had no change, or deteriorated in HbA1c, all achieved improvements in subcutaneous adiposity, but obtained mixed results for DBP changes. Furthermore, of the four participants in the MICT+RT group that had experience limited HbA1c change, each achieved improvements in other microvascular complication markers (one for postural stability, one for HRV, and two for uACR). However, of the seven participants in the HIIT+RT group that either had no change, or deteriorated in HbA1c, five remained unresponsive or deteriorated further in the microvascular complication markers. However, it must be noted that within such a study sample (i.e., morbidly obese men with moderate duration T2D), the maintenance of a function/marker can be considered as a favorable intervention outcome and should not be purely interpreted as “unresponsive.”

### Six month follow-up assessment

In comparison to baseline, both groups significantly improved their habitual physical activity during the 6-month follow-up phase (Table 2). During the follow-up assessment participants were scored on their adherence to regularly maintaining their training during the 6-month phase. While the MICT+RT adherence score was not significantly different to the HIIT+RT adherence score (*P* = 0.19), the MICT+RT group's mean score was 8.3 ± 3.7 [with one participant (9.1%) not continuing with any further training] and the HIIT+RT group's score 5.7 ± 5.4 [with five participants (45.5%) not continuing with any further training]. Nevertheless, both groups maintained their VO_2_max improvements achieved during the 12-week intervention. Although further study incorporating more regular and objective measures, which limit the impact of affecting external validity, are warranted, the medium-term durability of improved fitness is noteworthy in such a study sample.

During the 6-month follow-up, while both groups were able to maintain their overall habitual nutrition changes, both groups failed to maintain their refined carbohydrate score (Table 2). Additionally, both groups had changes to antihyperglycaemic medication usage. To elaborate, the MICT+RT group had one participant increase his daily insulin by 5.0 units, however two participants decreased by 4.0 and 2.0 units, respectively. Further, one MICT+RT participant was placed on Metformin and another had his sulfonylurea (Gliclazide) increased, but, of note, the MICT+RT participant who achieved partial remission remained as such. However, in the HIIT+RT group one participant was placed on Metformin and four participants using insulin had their daily dosage increased by an average of 9.5 units.

Importantly, at follow-up both groups significantly maintained the adiposity improvements of waist girth (Table 3) and subcutaneous adiposity (Figure 2C). However, both groups but failed to experience significant durability in their intervention achievements of improved HbA1c and HRV. On completion of the follow-up phase, both BMI and 10 g monofilament testing in both groups had reached significant change beyond baseline (Tables 3, 5, respectively), though BP, TG, HDL, hs-CRP, and postural stability remained indifferent to baseline values. The reduction in uACR was further compounded by the withdrawal of the participant diagnosed with congestive heart failure and a urine result that was not returned from the local hospital (both study participants having elevated concentrations at baseline).

Comparisons of effect durability to prior HIIT studies in people with T2D are limited as the only study to report a follow-up analysis was the pilot study by Hollekim-Strand et al. (25). At a 9-month follow-up (successive to a 12-week structured HIIT but unsupervised MICT intervention) the primary finding related to diastolic function (early diastolic tissue velocity) maintained an improvement for HIIT, but for MICT regressed during their follow-up period. Their interventions' initial small, yet significant, waist circumference improvements were retained for HIIT but diminished for MICT, and while the significant improvement in VO_2_peak for HIIT was sustained, the improvement in HbA1c for HIIT was not sustained (while the follow-up measure of HbA1c for MICT was not reported).

As our study only determined the short- and medium-term efficacy of either MICT+RT or HIIT+RT on markers of cardiometabolic risk and not on hard clinical end-points (e.g., myocardial infarction, lower limb amputations), studies with substantially longer follow-up periods are required to compare the impact of such interventions.

### Summary

Both training modalities significantly improved HbA1c, subcutaneous adiposity, HRV, and aerobic capacity during the 12-week intervention in the study's middle-aged obese men with moderate-duration T2D. Furthermore, adiposity and aerobic capacity were significantly maintained in both groups at the 6-month follow-up. As such, structured CV and RT training that progresses the CV component to either MICT or HIIT provides men living with T2D comparable glycaemic, cardiometabolic risk, and microvascular complication marker benefits.

#### Limitations

Both exercise groups had participants being prescribed multiple medications including metformin, beta-blockers and statins. For example, the HIIT+RT group had nine participants (75%) using statins and the MICT+RT group five participants (46%). Until well-designed clinical trials can determine the effect of statin therapy on muscle damage and reduced aerobic fitness benefits (92), the full extent to which statins impact cardiometabolic responses, particularly in people living with T2D, will be unknown. All participants underwent pre-exercise screening thereby limiting the inference of responses to the wider T2D population. During the 9 month study period, general practitioners' care for each participant resulted in antihyperglycaemic medication modifications in both groups. Although the changes in medication and habitual diet and physical activity were monitored, the extent to which these, alone or in combination, impacted our study's finding remains unclear. Subcutaneous adiposity was determined by skinfold thickness measurements. BP was measured manually, so the mean reading of four measures, over two non-consecutive days, was recorded for analysis in order to enhance reliability. During our 12-week progressive intervention study, the advanced stage was only applied for 5 weeks which may have limited the impact of the training stimuli. As each participant only experienced one type of CV modality, the PACES score can only be interpreted as affinity to exercise in general, and not as a preference to any one particular modality. In our study no blinding was possible as the lead researcher conducted all the assessments (except for the blood samples), supervised all the intervention sessions and captured and interpreted the data. In addition, due to nature of MICT and HIIT sessions both participants and assisting EPs were aware of the intervention modality. Although there was no attrition during the intervention phase, during the follow-up phase the one participant with pre-existing atrial fibrillation was diagnosed with congestive heart failure and withdrew from the study. Quantitative monitoring of participant adherence and compliance of training during the 6-month independent training phase was intentionally omitted so as to enhance external validity, but non-invasive monitoring technologies are required to observe, more quantitatively and qualitatively, the adoption of physical activity by participants after the introduction of an exercise intervention.

#### Strengths

The study included men more advanced in their T2D pathology (as indicated by their moderate-duration T2D, higher degree of obesity and medication usage) than those involved in previous studies. Our study provided a head-to-head comparison of a HIIT+RT modality to a modality recommended by multiple guidelines (i.e., MICT+RT) proposed by several expert groups (9, 93). The level of supervision by qualified EPs throughout the intervention stages allowed for intensity goals to be safely met within each session. Employing the method of minimization after both enrolment periods (Figure 1) assisted in enhancing internal validity and, notwithstanding the low sample size, the results of our study are relevant to all EPs working with men living with T2D in commercial settings, as such patients typically present with co-morbidities and multiple prescribed medications. Our study documented the exaggerated responses and precautionary respite employed. Data was provided at both group and participant level leading to a warning against classifying individuals simply as a “non-responder” to exercise without being specific about the nature of the specific response of interest. Additionally, our study included a 6-month follow-up assessment essential to establishing the potential for the sustained benefits of exercise.

#### Future research

Additional studies, using adequately powered designs incorporating increased training variables (i.e., intervention duration, training frequency, and/or session length), are required to determine the associations between baseline values and changes in diabetic complications and to determine whether magnitude of change in general health and fitness parameters (VO_2_max, strength, and adiposity) are related to specific macro- and microvascular function outcomes.

## Conclusions

Our study hypothesis of HIIT+RT affecting greater HbA1c reductions in men was rejected. Beyond improvements in aerobic capacity, both training modalities elicited similar benefits on HbA1c, adiposity and HRV in men living with T2D. In addition, during the intervention, study participants in both MICT+RT and HIIT+RT experienced favorable reductions to their hyperglycaemic medication usage. Furthermore, our study reported the inter-individual variability of change evident in both groups, the exaggerated acute physiological responses that occurred during both interventions as well as the incidence of precautionary respite afforded in such a study sample. Health practitioners have a greater understanding of the impact of two exercise modalities on cardiometabolic markers in men with moderate duration T2D.

In a group of middle-aged obese men living with T2D for a moderate duration, the completion of a structured 12-week intervention combining RT with either MICT or HIIT indicated that HIIT+RT is equivalent to MICT+RT for improving fitness, HbA1c, subcutaneous adiposity, HRV and distal foot sensation over the short and/or medium term. This indicates that the current guidelines are efficacious and exercise professionals can be confident including MICT+RT into their intervention strategies. In order to reduce hyperglycaemia, and prevent further deterioration in cardiometabolic risk and microvascular complication markers (in the short- and medium-term), future strategies that integrate the adoption and maintenance of physical activity as a cornerstone in the optimal treatment of T2M for men should include either structured MICT, or HIIT, combined with RT.

## Datasets

The raw data supporting the conclusions of this manuscript will be made available by the authors, without undue reservation, to any qualified researcher.

## Author contributions

SW, LD, CZ, and NH contributed to the conception of the work and along with RB all authors revised and approved the final manuscript. SW acquired and collated the data and wrote the first draft of the manuscript. SW, RB, and NH analyzed and interpreted the data. SW is the guarantor of the work and, as such, has full access to all the data in the study and takes responsibility for the integrity of the data and the accuracy of the data analysis.

### Conflict of interest statement

The authors declare that the research was conducted in the absence of any commercial or financial relationships that could be construed as a potential conflict of interest.
