# Supplementary material for: High-Intensity Interval Training Is Equivalent to Moderate-Intensity Continuous Training for Short- and Medium-Term Outcomes of Glucose Control, Cardiometabolic Risk, and Microvascular Complication Markers in Men With Type 2 Diabetes
Source: Front Endocrinol (Lausanne). 2018 Aug 28;9:475. doi: 10.3389/fendo.2018.00475 (PMC6120973; doi:10.3389/fendo.2018.00475)
Supplement: Supplementary file 1 [file Data_Sheet_1.docx]

**Appendix 1:** The penalty point allocation per check-box response for the 2008/09 New Zealand Adult Nutrition Survey (NZANS).

|  |  | Question No: | Guideline |  | Check- box: 1 | Check-box: 2 | Check-box: 3 | Check-box: 4 | Check-box: 5 | Check-box 6 | *Category Score* |
| --- | --- | --- | --- | --- | --- | --- | --- | --- | --- | --- | --- |
| Smoking | |  |  | **Input number of cigarettes smoked per day** | | | | | | | **30** |
|  | Cigarettes per day | 33 | No smoking |  |  | | | | | |  |
| Alcoholic drinks per week | | |  | **Product of penalties from questions 36 and 37** | | | | | | | **30** |
|  | Drink frequency | 36 | Less often |  | 0.5 | 1 | 3 | 4 |  |  |  |
|  | Drinks each occasion | 37 | Lower |  | 1.5 | 3 | 4.5 | 6 | 7.5 |  |  |
| Fast-foods per week | |  |  | **Sum of penalties from questions 11, 21 and 22** | | | | | | | **30** |
|  | Battered fish | 11 | Lower |  | 0 | 2 | 4 | 6 | 8 | 10 |  |
|  | Hot chips | 21 | Lower |  | 0 | 2 | 4 | 6 | 8 | 10 |  |
|  | Take-aways | 22 | Lower |  | 0 | 2 | 4 | 6 | 8 | 10 |  |
| Refined carbohydrates | |  |  | **Sum of penalties from questions 23 to 25** | | | | | | | **50** |
|  | Fruit juices | 23 | Lower |  | 0 | 2 | 4 | 6 | 8 | 10 |  |
|  | Soft drinks | 24 | Lower |  | 0 | 2 | 4 | 6 | 8 | 10 |  |
|  | Sugar via hot drinks | 24a | Lower |  | Input number of teaspoons of sugar via hot beverages | | | | | |  |
|  | Lollies/candy | 25 | Lower |  | 0 | 2 | 4 | 6 | 8 | 10 |  |
| General food quality | |  |  | **Sum of penalties from the following questions** | | | | | | | **60** |
|  | Breakfast frequency | 1 | Higher |  | Input number of days breakfast skipped per week | | | | | | |
|  | Slices of bread | 2 | Lower |  | 0 | 0.5 | 1 | 3 | 5 | 7 |  |
|  | Bread’s grain quality | 3 | Higher |  | 4 | 3 | 2 | 1 | 1.5 |  |  |
|  | Eat Meat (unprocessed) | 5 | 1-2 x / week |  | 2 | 1 | 0 | 1 | 2 | 4 |  |
|  | Eat Chicken | 6 | 2-4 x / week |  | 2 | 1 | 0 | 0 | 1 | 2 |  |
|  | Eat Fish (not battered) | 10 | 2-4 x / week |  | 2 | 1 | 0 | 0 | 0.5 | 1 |  |
|  | Remove meat fat | 7 | More often |  | 4 | 3 | 2 | 1 | 0 |  |  |
|  | Remove chicken skin | 8 | More often |  | 4 | 3 | 2 | 1 | 0 |  |  |
|  | Eat processed meat | 9 | Less often |  | 0 | 1 | 2 | 3 | 4 | 5 |  |
|  | Eat canned fish | 12 | More often |  | 5 | 4 | 3 | 2 | 1 | 0 |  |
|  | Fruit consumption | 13 | More often |  | 5 | 4 | 3 | 2 | 1 | 0 |  |
|  | Vegetable consumption | 14 | More often |  | 5 | 4 | 3 | 2 | 1 | 0 |  |
|  | Salt adding | 18 | Less often |  | 0 | 1 | 2 | 3 | 4 |  |  |
|  | Choose low fat option | 19 | More often |  | 4 | 3 | 2 | 1 | 0 |  |  |
|  | Choose low salt option | 20 | More often |  | 4 | 3 | 2 | 1 | 0 |  |  |
| TOTAL | |  |  | **SUM OF THE FIVE DOMAINS** | | | | | | | **200** |
